# Characterization of Extended-Spectrum β-Lactamase-Producing *Escherichia coli* in Animal Farms in Hunan Province, China

**DOI:** 10.3390/microorganisms12040653

**Published:** 2024-03-26

**Authors:** Ning Xiao, Yujuan Li, Hongguang Lin, Jie Yang, Gang Xiao, Zonghan Jiang, Yunqiang Zhang, Wenxin Chen, Pengcheng Zhou, Zhiliang Sun, Jiyun Li

**Affiliations:** 1 College of Veterinary Medicine, Hunan Agricultural University, Changsha 410128, China; xn15873416884@163.com (N.X.); jieyang1@foxmail.com (J.Y.); xiaogang2020@stu.hunau.edu.cn (G.X.); jiangzonghan0622@163.com (Z.J.); yunqiangzhang2023@163.com (Y.Z.); chenwenxin2022@foxmail.com (W.C.); 19373405521@163.com (P.Z.); 2 Hunan Engineering Technology Research Center of Veterinary Drugs, Hunan Agricultural University, Changsha 410128, China; li_yujuan1001@163.com (Y.L.); linhongguang@stu.hunau.edu.cn (H.L.); 3 Institute of Biopharmaceutical and Health Engineering, Tsinghua Shenzhen International Graduate School, Tsinghua University, Shenzhen 518055, China

**Keywords:** ESBL, *Escherichia coli*, *bla*_CTX-M_, Hunan Province, animal farms

## Abstract

Multi-drug resistance of bacteria producing extended-spectrum β-lactamase (ESBL) is a public health challenge. Thus, this study aimed to investigate the antimicrobial susceptibility of ESBL-producing *Escherichia coli* (ESBL-EC) in Hunan Province, China. A total of 1366 fecal samples were collected from pig, chicken, and cattle farms over a six-year period, which were assessed using strain isolation, 16S rRNA identification, polymerase chain reaction, drug sensitivity testing, whole-genome sequencing, and bioinformatics analysis. The results showed an overall prevalence of 6.66% for ESBL-EC strains, with ESBL positivity extents for pigs, chickens, and cattle isolates at 6.77%, 6.54%, and 12.5%, respectively. Most ESBL-EC isolates were resistant to cefotaxime, tetracycline, and trimethoprim-sulfamethoxazole; however, all the isolates were susceptible to meropenem, with relatively low resistance to amikacin and tigecycline. Various multi-locus sequence types with different origins and similar affinities were identified, with ST155 (*n* = 16) being the most common subtype. Several types of resistance genes were identified among the 91 positive strains, with beta-lactamase *bla*_CTX-M-55_ being the most common ESBL genotype. IncFIB was the predominant plasmid type. Widespread use of antibiotics in animal farming may increase antibiotic resistance, posing a serious threat to the health of farmed animals and, thus, to human food security and health.

## 1. Introduction

Since the discovery of penicillin by Alexander Fleming in 1929, numerous antimicrobial agents have been developed. However, the widespread excessive prescription and irresponsible use of antibiotics have resulted in a surge of drug-resistant strains [1]. Antibiotics have always been the key to treating bacterial infections in humans and animals; however, bacterial resistance has hindered the treatment process, threatening public health security [2]. Thus, antimicrobial resistance (AMR), particularly the resistance to common antibiotics by bacterial pathogens that cause human diseases, is a pressing issue that affects global health and development [3].

Antimicrobial resistance and antibiotic resistance genes (ARGs) present multi-faceted challenges that have become some of the greatest threats to human health in the 21st century [4]. One prominent manifestation of antibiotic resistance is observed in extended-spectrum β-lactamases (ESBL), which can inactivate cephalosporins, monobactams, and penicillin [5]. Most broad-spectrum β-lactamases present with three genotypes: TEM, SHV, and CTX-M, with hundreds of widely spread variants [6]. The β-lactamase *bla*_TEM-1_ type ESBL resistance gene was first isolated in Athens, Greece, in 1963 [7], and ESBL-producing Enterobacteriaceae were first reported in Germany in 1983 [8], after which their prevalence (especially in *Escherichia coli* [9] and *Klebsiella pneumoniae* [10]) increased dramatically. Notably, resistant bacteria can spread from animals to humans, and vice versa, through direct contact, the food chain, or the environment [11]. In the late 1990s, *bla*_CTX-M_ replaced *bla*_TEM_ and *bla*_SHV_ as the dominant ESBL genotype worldwide [12].

The emergence of ESBL-producing *Escherichia coli* (ESBL-EC) can be attributed primarily to the selective pressure of antibiotics used in human and veterinary medicine, including that associated with the administration of antibiotics in animal feeds. *Escherichia coli* can hydrolyze some antibacterial drugs [13] and target cephalosporins, including cefotaxime, ceftriaxone, ceftazidime, and cefepime [14]. Antibiotic hydrolysis by bacteria producing β-lactamases (penicillinase, cephalosporinase, among others) affects treatment efficacy [15] for conditions that include urinary tract infections, intra-abdominal infections, respiratory infections, and bacteremia, increasing the probability of morbidity and mortality [11]. Globally, 700,000 deaths occur annually owing to drug-resistant bacterial infections [16]; this estimate is projected to increase to 10 million cases in 2050, costing an estimated USD 100 trillion in productive output loss [17]. The pressing issue of drug resistance is leading to significant challenges and economic losses for humans in the realms of medical clinics, veterinary clinics, and food safety [18].

The global usage of antibiotics for veterinary purposes is twice that for human care [19]. In 2017, China was the largest consumer of veterinary antibiotics [20], constituting 45% of global usage; this proportion is expected to persist through 2030 (43%) [21]. The four most widely administered classes of veterinary antimicrobials in China in 2019 were, in order of usage, tetracyclines, peptides, beta-lactams, and macrolides (https://www.moa.gov.cn/gk/sygb/ (accessed on 30 October 2023)). Selection pressures associated with antibiotic use tend to favor pathogens that show early signs of resistance [22], creating urgency around studies that examine the prevalence of ESBL-EC. A Canadian combined surveillance study conducted between 2012 and 2017 showed that ESBL-producing *Salmonella* and *E. coli* were present in humans, as well as in animals intended for human consumption. The prevalence of confirmed ESBL-EC in agrifood samples during this period ranged between 0.5% and 3%, with a higher frequency observed in samples from broiler farms [23]. In Thailand, studies reported a high prevalence of broad-spectrum cephalosporin-resistant *Escherichia coli* (ESC-) and colistin-resistant *Escherichia coli* (Col-R-Ec), and found that isolation of ESBL-EC (especially CTX-M-55) was higher in swine farmers than in non-swine farmers [24]. A high percentage of ESBL-EC was found in swine-related samples from Korea, and isolates carried common clinical ESBL genotypes [25]. However, only a few studies have been reported on the prevalence of ESBL-EC in Chinese animal farms. Therefore, in this study, we aimed to investigate, for the first time, the prevalence of ESBL-EC in animal farms in Hunan Province, China, with the aim of helping to design interventions to reduce and reverse AMR.

## 2. Materials and Methods

### 2.1. Sample Collection

In China, the National Action Plan for Curbing Bacterial Resistance (2016–2020) (https://www.who.int/publications/m/item/china-national-action-plan-to-contain-antimicrobial-resistance-(2016-2020) (accessed on 1 July 2015)) assists in establishing and improving the monitoring network for veterinary antimicrobial drug application and bacterial resistance of animal origin, covering farms (households) with different breeding methods and species, and gathering epidemiological data on bacterial resistance of animal origin. In this context, in order to improve the database of animal-derived drug resistance genes in Hunan Province, we randomly sampled both intensive and small-scale pig, chicken, and cattle farms in 12 urban areas of Hunan Province from 2016 to 2021. Random samples were taken annually at the relevant animal farms with which the unit cooperated, with a total of 1366 samples taken. The sample numbers for each year were as follows: 2016 (*n* = 237), 2017 (*n* = 256), 2018 (*n* = 116), 2019 (*n* = 254), 2020 (*n* = 248), and 2021 (*n* = 255). By species origin, the sample numbers were as follows: pigs (*n* = 502), chickens (*n* = 856), and cattle (*n* = 8).

The vast majority of farms sampled each year were the same as in previous years. The origin, sampling time, species origin, and animal health status of the samples were recorded during sampling. Animal feces samples were collected with sterile swabs. These swabs were then placed in Eppendorf (EP) tubes containing Luria–Bertani (LB) broth, and then the EP tubes were placed in an ice box for preservation. All samples were maintained at 4 °C during transportation to the laboratory and were processed within 72 h of sampling.

### 2.2. Bacterial Isolation and Identification

The samples were inoculated on MacConkey agar plates with sterile inoculation rings and cultured overnight at 37 °C. Single colonies were then inoculated in 500 μL LB broth and cultured overnight at 37 °C. The 500 μL bacterial solution was centrifuged at 1000 rpm, the supernatant was discarded, and the precipitate was washed with 100 μL of sterile water. This washing procedure was repeated once. Subsequently, the solution was boiled in water at 100 °C for 10–15 min, removed and cooled on ice for 5 min, then centrifuged at 1000 rpm at 4 °C for 2 min. Thereafter, the supernatant was transferred to new sterile EP tubes and put into a 4 °C refrigerator for short-term storage or a −20 °C freezer for long-term storage [26]. Species identification was performed using 16S rRNA gene sequencing [27]. All strains were screened for the presence of ESBL genes (*bla*_CTX-M_, *bla*_TEM_, and *bla*_SHV_) using polymerase chain reaction (PCR) [28]. The 2× NG PCR MasterMix (Shanghai Huiling Biotechnology Co., Ltd., Shanghai, China) was used for PCR experiments. Known ESBL-EC-positive strains maintained in the laboratory were used for quality control during screening. For culturing strains, we used MacConkey agar, LB broth, LB agar, Mueller–Hinton agar, and Mueller–Hinton broth from Beijing LAND BRIDGE (Beijing, China).

### 2.3. Antimicrobial Susceptibility Testing

Susceptibility testing of ESBL gene-positive strains was performed using broth microdilution, according to International Organization for Standardization standard ISO 20776-1 (https://www.iso.org/standard/70464.html/, (accessed on 30 October 2023)), with twofold dilutions of cefotaxime, ceftazidime, gentamicin, amika-cin, ciprofloxacin, colistin, meropenem, tetracycline, tigecycline, and trimethoprim/sulfamethoxazole. The medium was cation-adjusted Mueller–Hinton broth. Tetracycline resistance breakpoints were referenced to Clinical and Laboratory Standards Institute (CLSI M100-S30) standards (https://clsi.org/media/3481/m100ed30_sample.pdf, (accessed on 30 October 2023).). Other antibiotic analysis results were interpreted according to the European Committee for Antimicrobial Susceptibility Testing (EUCAST) Minimum Inhibitory Concentrations (MIC) Interpretation Breakpoints Table (version 14.0; http://www.eucast.org, (accessed on 30 October 2023).). The strain concentration was 5 × 10^5^ CFU/mL. The incubation conditions were sealed panels, air, 35 ± 1 °C, 18 ± 2 h. *Escherechia coli* strain ATCC 25922 was used for quality control.

### 2.4. Whole-Genome Sequencing

DNA of ESBL isolates was extracted using the TIANamp Bacteria DNA kit (Tiangen Biotechnology Co., Ltd., Beijing, China) according to the manufacturer’s instructions [11]. Whole-genome sequencing was performed using Annoroad Gene Technology (Beijing, China) on a Nova Seq 6000 S4 sequencing platform using the Nova Seq 6000 S4 Reagent kit V1.5 [29]. For each *E. coli* isolate analyzed via whole-genome sequencing, a minimum of 100-fold coverage of the original reads was generated and collected [30].

### 2.5. Bioinformatics Analysis

The virulence genes and AMR genes of the strains were analyzed using the VirulenceFinder and ResFinder tools from the Center for Genomic Epidemiology (https://www.genomicepidemiology.org/ (accessed on 5 November 2023)). The thresholds for identification and annotation were 90% (%ID) and 60% (minimum length). We utilized seven housekeeping genes (adk, fumC, gyrB, icd, mdh, purA, and recA) for MLST identification [31]. Bacterial genome assembly was performed using SPAdes software (version 3.11) [32]. The relationships among ESBL-EC were assessed using core genome alignment, constructed using Parsnp [33] and the neighbor-joining method, and visualized using the online tool iTOL 6.5.7 [34]. The phylogenetic groups (A, B1, B2, D, E, and F) of the *E. coli* isolates were identified using previously described methods [35]. Minimum spanning trees of multi-locus sequence types (MLST) were generated for all sequence types (site and species origins) using phylogenetic inference and data visualization (PHYLOViZ Online) (https://online.phyloviz.net/index (accessed on 5 November 2023)).

## 3. Results

### 3.1. Strain Isolation and Characterization

A total of 1287 *E. coli* strains were isolated from 1366 samples. Screening for common β-lactam resistance genes (*bla*_CTX-M_, *bla*_TEM_, and *bla*_SHV_) using PCR revealed that only 91 isolates contained either the *bla*_CTX-M_ or *bla*_TEM_ genes, with no *bla*_SHV_ genes observed. These 91 isolates were further characterized as ESBL-EC-positive strains through 16S rRNA gene sequencing, representing an overall ESBL-EC prevalence of 6.66% (91/1366) (Table S1). The isolation proportions of ESBL-EC positive samples for the years 2016–2021 were 10.13% (24/237), 7.42% (19/256), 6.03% (7/116), 7.87% (20/254), 4.84% (12/248), and 5.10% (13/255), respectively. The overall prevalence of ESBL-EC strains was 6.66%, with ESBL positivity extents among pigs, chickens, and cattle isolates recorded as 6.77% (34/502), 6.54% (56/856), and 12.5% (1/8), respectively.

### 3.2. Antimicrobial Susceptibility Testing

More than 89% of the 91 ESBL-EC isolates were resistant to cefotaxime, tetracycline, and trimethoprim-sulfamethoxazole, as determined by MIC values (Table S2), surpassing the resistance breakpoints defined by the EUCAST (Table 1). Additionally, all ESBL-EC isolates were susceptible to meropenem and showed relatively low resistance levels to amikacin and tigecycline (Table 1). The results showed that most of the 91 isolates were concurrently resistant to two or more drugs from beta-lactams, aminoglycosides, quinolones, colistin, tetracycline, and trimethoprim-sulphonamide, reflecting the phenomenon of multi-drug resistance in some farms (Table S2).

The ESBL-EC-positive isolates also exhibited multi-drug resistance (Table 2). Among the 24 ESBL-EC positive isolates collected in 2016, resistance to trimethoprim-sulfamethoxazole was observed, with over 95% resistance to cefotaxime and tetracycline; however, all remained susceptible to meropenem and tigecycline. Conversely, among the 13 ESBL-EC positive isolates collected in 2021, all exhibited resistance to trimethoprim-sulfamethoxazole, with over 92% showing resistance to cefotaxime, while none were resistant to meropenem (Table 2). Throughout the six consecutive years, these ESBL-EC-positive isolates consistently showed over 70% resistance to cefotaxime and over 60% resistance to tetracycline and trimethoprim-sulfamethoxazole, and remained susceptible to meropenem (Table 2).

ESBL-EC isolates from pigs showed a significantly higher proportion of resistance to cefotaxime, ciprofloxacin, colistin, and tigecycline compared to those from chickens (Table 2). Conversely, isolates from chickens demonstrated a higher proportion of resistance to gentamicin, amikacin, tetracycline, and trimethoprim sulfamethoxazole than that exhibited by pigs (Table 2). All ESBL-EC isolates were sensitive to meropenem and exhibited relatively low proportions of resistance to amikacin, colistin, and tigecycline (Table 2).

### 3.3. Multi-Locus Sequence Types and Antibiotic Resistance Genes

**Figure 1:**
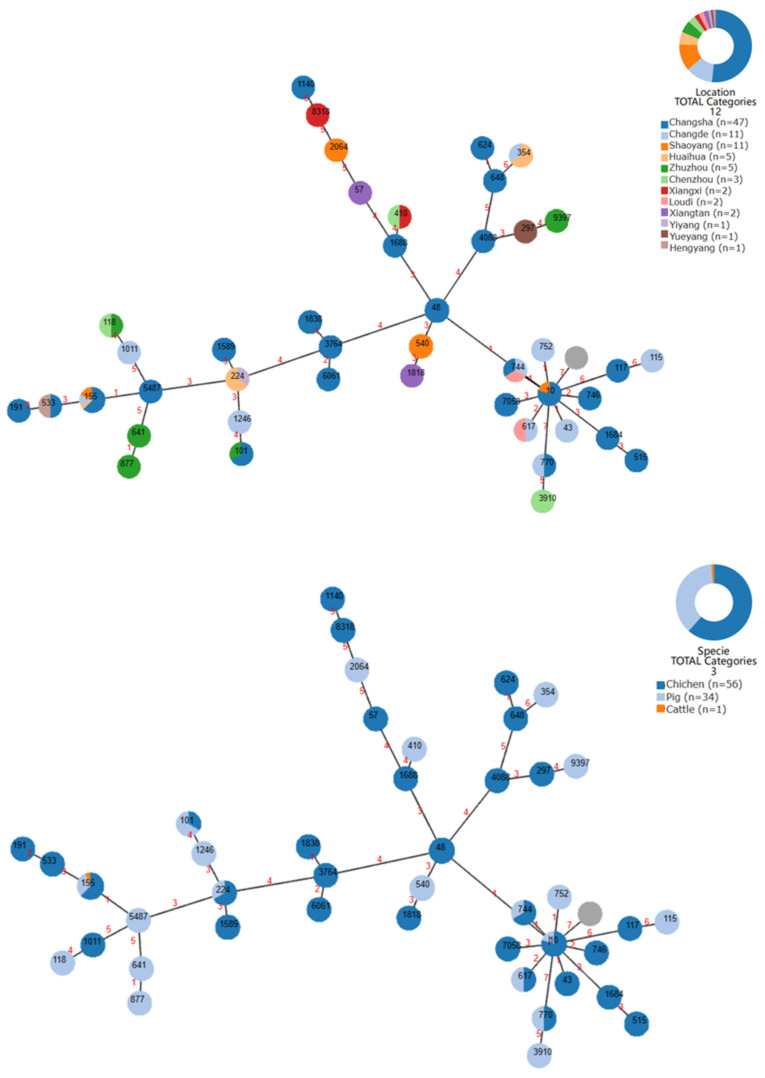
Minimum spanning tree of sampling site origin and species origin for 91 ESBL-EC-positive strains. Each node in the tree represents a sequence type (ST). The label of a node indicates the ST type. The color of the node indicates the sampling location or species contained in that ST type, and the intensity of the color indicates the proportion of the number of ESBL-EC-positive samples contained. The branch length between each node is proportional to the number/ST of different alleles among the seven multi-locus sequence type (MLST) genes between two connected nodes. Numbers between two nodes indicate absolute distances in terms of proximity of kinship.

Forty-three MLST types were identified using whole-genome sequencing (Figure 1). The most frequent ST types were ST155 (*n* = 16), ST48 (*n* = 8), ST540 (*n* = 6), ST10 (*n* = 5), ST101 (*n* = 3), ST224 (*n* = 3), ST354 (*n* = 3), ST744 (*n* = 3), and ST1589 (*n* = 3), with ST155 being the most common ST subtype (17.6%) among ESBL-EC isolates, including those from pigs (*n* = 5), chickens (*n* = 10), and cattle (*n* = 1). ST155 contained ESBL-EC isolates across a broad temporal range, present in every year except 2018, and demonstrated a wide geographic distribution, with isolates identified in the Changsha, Changde, Shaoyang, and Huaihua isolates.

**Figure 2:**
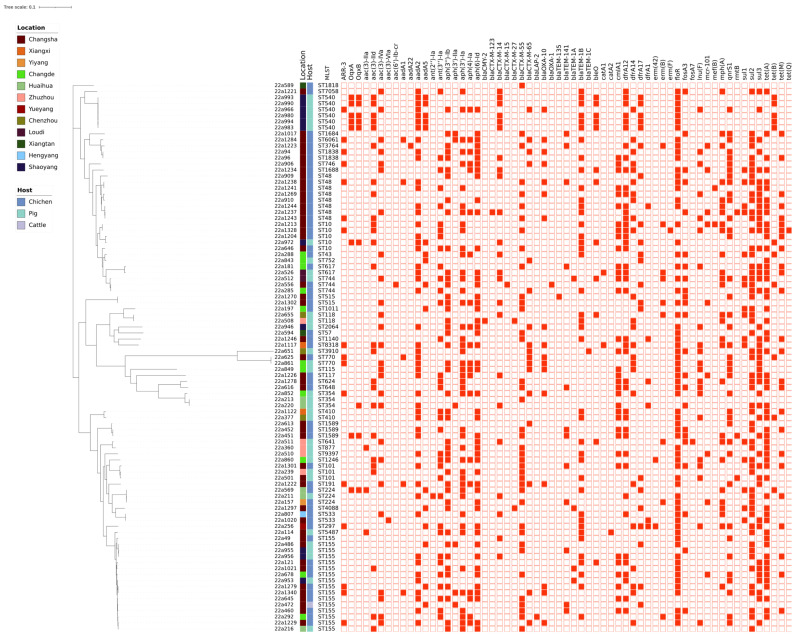
Phylogenetic tree illustrating the genetic relationships, MLST, and resistance genes of 91 ESBL-EC isolates from various cities and states in Hunan Province. This tree, constructed using core genomic single nucleotide polymorphism (SNP) data, is rooted at the midpoint. The city and state location origin and species origin of ESBL-EC-positive isolates are distinguished by the color of the name tag. The red heat map depicts the presence or absence of antimicrobial resistance (AMR) genes.

Among the 91 strains, genotypic analysis of the ARG in these ESBL-EC-positive isolates revealed 61 ARG for 10 types of antibiotics (β-lactams, aminoglycosides, macrolides, sulphonamides, quinolone, rifampicin, fosfomycin, tetracycline, chloramphenicol, and colistin) (Figure 2). A total of 6 β-lactam-resistant genes (*bla*_CTX-14_, *bla*_CTX-55_, *bla*_CTX-65_, *bla*_OXA-10_, *bla*_TEM-141_, and *bla*_CTX-1B_) were the most common ESBL-type ARGs among the 91 isolates. The 22a197 (ST1011) and 22a1223 (ST3764) strains harbored the highest numbers of resistance genes. Notably, eight strains—22a239 (ST101), 22a288 (ST43), 22a360 (ST877), 22a511 (ST641), 22a909 (ST48), 22a1229 (ST155), 22a1243 (ST48), and 22a1301 (ST101)—exhibited the presence of genes conferring resistance to β-lactams, aminoglycosides, macrolides, tetracyclines, sulphonamides, quinolones, and chloramphenicol. Resistance genes were detected in most phenotypically resistant isolates, and phenotypically sensitive isolates were also found to harbor associated resistance genes (Table 3).

### 3.4. Plasmid Replicon and Evolutionary Analysis

Despite originating from varied sources and collection sites, the 91 ESBL-EC isolates shared common plasmid replicon types, with Col, IncF, and IncX being the dominant ones. These plasmids, carrying ESBL resistance genes, play a crucial role in gene transmission (Table S1). Changsha, located at the heart of Hunan Province, emerged as an epicenter of positivity in our sample collection, accounting for 51.65% (*n* = 47) of all positive samples. The evolutionary tree revealed that, among the 91 ESBL-EC isolates sequenced, 61 ARGs were identified, including *bla*_CTX-M-55_, *floR*, *sul2*, *tet*(A), *aadA2*, *aph*(6)-*Id*, *sul3*, *aph*(3′)-*Ia*, *aph*(3″)-*Ib*, *dfrA12*, and *bla*_TEM-1B_ (Figure 2).

## 4. Discussion

China leads the world in pork production, ranks second in broiler production, and ranks third in beef production [36]. Additionally, it is also the largest consumer of antibiotics globally [37]. The gross output value of veterinary drugs manufactured in China correlates with the average counts of ARG over various time periods; however, no such correlation exists with the average count of plasmid-associated replicons [38]. The increased use of antibiotics contributes to the spread of AMR. Liu et al. conducted a study from 2015 to 2019, collecting fecal samples (*n* = 344) from livestock farms in Guangdong, Shandong, Xinjiang, and Heilongjiang provinces in China. They reported ESBL-EC prevalences were 78.6% (110/140) in chickens, 70.7% (58/82) in cattle, and 75.4% (92/122) in pigs [39]. Similarly, Wu et al. collected 341 chicken cloacal swabs from Guangdong, Shanghai, and Shaanxi in China from 2008 to 2014. They found that the prevalence of ESBL-EC increased from 23.8% in 2008 to 57.0% in 2014 [40]. The positivity proportion of chicken-derived ESBL-EC in China, as reported in the study, was lower than that reported in previous studies. This discrepancy in the findings indicates that the “one health” policy may possibly be well-implemented in poultry farms in Hunan Province [41,42].

Based on their MIC values, most of the strains identified in this study were not resistant to amikacin, tigecycline, or meropenem, aligning with the drug usage habits of poultry farms. However, three strains—22a49, 22a377, and 22a1226—tended to be resistant to aminoglycoside antibiotics, a phenotype explainable by the presence of corresponding resistance genes. Notably, among the 16 ESBL-EC-positive strains of ST155, 22a1279 and 22a1340 originated from the same chicken farm. Moreover, 22a953, 22a955, and 22a956 were collected from the same pig farm. In addition, the fact that 22a213 and 22a220 were vertically transmitted over many generations and still belonged to the ST354 phenotype suggests that these two strains may have evolved from the same strain (Figure 2). Our findings also identified 17 different ARGs in strain 22a1229 (ST155), displaying simultaneous phenotypic resistance to beta-lactams, aminoglycosides, quinolones, colistin, tetracycline, and trimethoprim/sulphonamide. The emergence of these multi-drug-resistant phenotypes suggests that ESBL genes coexist with other resistance genes in *E. coli* strains, expanding their resistance profiles [43] and potentially limiting the efficacy of antibiotics in industrial farming. This study underscores the presence of multi-drug-resistant *E. coli* in animal farming, highlighting the critical challenge of ESBL-EC.

The whole genomes of the identified ESBL-EC-positive strains were sequenced and used for phylogenetic trees and other bioinformatics analyses. Phylogenetic tree analysis showed that the 91 isolates carrying ESBLs were genetically diverse, categorized into 43 different STs. Although some of these isolates were from the same locality, their STs varied, indicating the presence of a widespread ESBL gene among different ST subtypes. Among them, the *bla*_CTX-M_ phenotype is the major ESBL-encoding gene in ESBL-EC, with 87.9% of isolates carrying the *bla*_CTX-M_ gene, which is slightly higher than the 86% previously reported in Chinese flocks [44] but lower than the 96.9% reported in human isolates [45]. In this study, six subtypes of genotypes—*bla*_CTX-M_-14, -15, -27, -55, -65, and -123—exhibited prevalences of 25.3%, 1.1%, 2.2%, 42.9%, 18.7%, and 1.1%, respectively. In the present study, we identified *bla*_CTX-M-55_ as the predominant genotype of *bla*_CTX-M_, whereas *bla*_CTX-M-14_ was also identified as the predominant ESBL subtype in patients with pneumonia [46]. The prevalence of *bla*_CTX-M-55_ was approximately equal to that of *bla*_CTX-M-14_ and *bla*_CTX-M-65_. However, it is important to note that *bla*_CTX-M-123_ had not been reported in human clinical bacteria in China before 2012 [47], and this subtype has rarely been reported since.

In this study, the *bla*_CTX-M-123_ subtype was identified in strain 22a1226, which is a chicken-derived ESBL. Interestingly, *bla*_CTX-M-14_ and *bla*_CTX-M-123_ were both present in strain 22a1226; *bla*_CTX-M-14_ and *bla*_CTX-M-55_ were both present in strain 22a1284; and *bla*_CTX-M-55_ and *bla*_CTX-M-65_ were both present in strain 22a1226, which greatly increased the risk of plasmid-mediated transmission of drug resistance. The degree of plasmid homology observed across porcine, avian, and cattle populations suggests its extensive dissemination among diverse species and an urgent need for the spread of surveillance and control. Reducing AMR is paramount to global health and food safety and security.

Previously, there have been few reports on three drug-resistant genes, *bla*_CTX-M-27_, *bla*_CTX-M-65_, and *bla*_CTX-M-123_, all of which have appeared for the first time in Hunan Province. In this study, for the 17 drug-resistant strains carrying *bla*_CTX-M-65_, a cluster of isolated homozygous overlapping strains was compared with the polycyclic sequence of the reference plasmid, showing that six of the isolated strains were in complete agreement, and most of the other strains were also in agreement. Moreover, strains 22a1270 and 22a1302, both categorized as ST515 and sharing identical source information, as well as possessing nearly identical ARGs, exhibited variations in phenotype. A hypothesis was formulated, suggesting that the presence of this gene may be the underlying cause of these observed phenotypic differences.

Over the years 2016–2021, 91 animal-derived ESBL-EC isolates consistently exhibited resistance to cefotaxime at an extent exceeding 70%, as well as to tetracycline and trimethoprim-sulfamethoxazole at an extent consistently greater than 60%, indicating a high frequency of use of these three drugs in animal farms in Hunan Province. Furthermore, the resistance extent to gentamicin consistently surpassed 40%, suggesting substantial usage of gentamicin in these settings. It is evident that the resistance extent of ESBL-EC isolates to these ten drugs was lower in 2017 than in 2016, likely attributable to the implementation of the National Action Plan for Containment of Bacterial Drug Resistance issued by the Chinese governmental department in 2016, indicating proactive measures undertaken by all animal farms in Hunan Province. However, some drugs exhibited increases in resistance extent in 2018 or 2019, possibly linked to the outbreak of African swine fever and other livestock diseases in China in 2018. Bacterial resistance of animal origin is a matter of significant international concern. Following the discovery of the plasmid-mediated colistin resistance gene, *mcr-1*, the Chinese government banned the use of colistin as an animal growth promoter in April 2017. Consequently, the resistance extent of colistin decreased significantly from 29.17% in 2016 to 10.53% in 2017. However, the persistence of resistance in 2019 suggests that some animal farms continue to use colistin in violation of the law. Given that meropenem is primarily used as an anti-infective drug for treating infections in humans, the Chinese Veterinary Drug Administration Regulations prohibit its use in animals (https://www.nahs.org.cn/gk/zcjd/202104/t20210407_375367.htm (accessed on 5 November 2023)). Consequently, none of the animal-derived ESBL-EC isolates showed resistance to meropenem.

## 5. Limitations

First, this study focuses on the prevalence of ESBL-EC in the Hunan province, necessitating the aggregation of sample sources at a city and state level. Our organization collected samples from visiting partner companies. While we have information on the size of some livestock farms, specific details such as exact numbers, antibiotic treatments, and animal infections remain strictly confidential.

Second, operational challenges, the emergence of new partner enterprises, the COVID-19 pandemic, and African swine fever constrained our ability to visit some partner enterprises’ animal farms. Consequently, we could not ensure that all animal farms were inspected according to the planned annual sampling program.

## 6. Conclusions

The widespread presence of ESBL-EC in animal farms in Hunan Province, China, suggests that the common use of antibiotics in industrial farming may promote AMR, threatening human and animal health and potentially resulting in economic losses. Phylogenetic analyses revealed a consistently high prevalence of ESBL-EC in farm animal isolates, thereby highlighting the need to increase ESBL-EC monitoring in clinical veterinary medicine and farming. Healthy livestock is required in order to ensure global food safety and security, and efforts must be undertaken to prevent and treat disease among animals, including the use of antibiotics. Since antimicrobials are selected for specific resistance genotypes, the coexistence and cotransformation of ARG must be considered when designing strategies to reduce AMR, which may require targeting different classes of antimicrobials.

## Figures and Tables

**Table 1:** Resistance phenotypes of 91 extended-spectrum β-lactamase-producing *Escherichia coli* (ESBL-EC) strains.

Antibiotic Agent	Antibiotic Concentration Range (mg/L)	Breakpoint Interpretive Criteria (mg/L)			Results in Percentage (%)		
		S	I	R	S	I	R
Cefotaxime	0.015–128	≤1	2	>2	7 (7.69%)	2 (2.20%)	82 (90.11%)
Ceftazidime	0.03–128	≤1	2–4	>4	28 (30.77%)	15 (16.48%)	48 (52.75%)
Gentamicin	0.125–128	≤2	-	>2	32 (35.16%)	0	59 (64.84%)
Amikacin	0.25–128	≤8	-	>8	88 (96.70%)	0	3 (3.30%)
Ciprofloxacin	0.002–128	≤0.25	0.5	>0.5	28 (30.77%)	8 (8.79%)	55 (60.44%)
Colistin	0.125–64	≤2	-	>2	78 (85.71%)	0	13 (14.29%)
Meropenem	0.004–4	≤2	4–8	>8	91 (100%)	0	0
Tetracycline	0.25–128	≤4	8–16	>16	5 (5.49%)	4 (4.40%)	82 (90.11%)
Tigecycline	0.015–16	≤0.5	-	>0.5	89 (97.80%)	0	2 (2.20%)
Trimethoprim/Sulfamethoxazole	0.25/4.75–32/608	≤2/38	4/76	>4/76	7 (7.69%)	3 (3.30%)	81 (89.01%)

**Table 2:** Number of strains with drug-resistant phenotypes and differences in resistance phenotypes in pigs and chickens.

Antibiotic Agent	2016 (*n* = 24)	2017 (*n* = 19)	2018 (*n* = 6)	2019 (*n* = 17)	2020 (*n* = 12)	2021 (*n* = 13)	Pig (*n* = 34)	Chicken (*n* =56)	Cattle (*n* = 1)
Cefotaxime	23 (95.83%)	14 (73.68%)	6 (100%)	17 (100%)	10 (83.33%)	12 (92.31%)	31 (91.18%)	50 (89.29%)	1 (100%)
Ceftazidime	16 (66.67%)	5 (26.32%)	2 (33.33%)	12 (70.59%)	7 (58.33%)	6 (46.15%)	18 (52.94%)	29 (51.79%)	1 (100%)
Gentamicin	19 (79.17%)	12 (63.16%)	5 (83.33%)	9 (52.94%)	5 (41.67%)	9 (69.23%)	22 (64.71%)	37 (66.07%)	0
Amikacin	1 (4.17%)	0	0	0	1 (8.33%)	1 (7.69%)	1 (2.94%)	2 (3.57)	0
Ciprofloxacin	14 (58.33%)	7 (36.84%)	1 (16.67%)	13 (76.47%)	10 (83.33%)	10 (76.92%)	22 (64.71%)	32 (57.14%)	1 (100%)
Colistin	7 (29.17%)	2 (10.53%)	0	2 (11.76%)	0	2 (15.38%)	5 (14.71%)	8 (14.29%)	0
Meropenem	0	0	0	0	0	0	0	0	0
Tetracycline	23 (95.83%)	18 (94.74%)	4 (66.67%)	15 (88.24%)	11 (91.67%)	11 (84.62%)	30 (88.24%)	51 (91.07%)	1 (100%)
Tigecycline	0	0	0	1 (5.88%)	0	1 (7.69%)	2 (5.88%)	0	0
Trimethoprim Sulfamethoxazole	24 (100%)	14 (73.68%)	4 (66.67%)	15 (88.24%)	11 (91.67%)	13 (100%)	30 (88.24%)	50 (89.29%)	1 (100%)

**Table 3:** Comparison of drug-resistant phenotypes and genotypes of 91 ESBL-EC strains.

Antibiotic	Phenotype: Susceptible		Phenotype: Resistant	
	Genotype: Resistant	Genotype: Susceptible	Genotype: Resistant	Genotype: Susceptible
Beta-lactam	9	0	82	0
Aminoglycoside	31	1	55	4
Quinolone	21	15	27	28
Colistin	6	72	3	10
Tetracycline	7	2	60	22
Trimethoprim/Sulphonamide	10	0	78	3

Note: Phenotypic sensitivities are categorized as “S” (susceptible, standard dosing regimen) and “I” (susceptible, increased exposure). Cefotaxime, ceftazidime, and meropenem are beta-lactamases. Isolates resistant to ceftazidime and meropenem are also resistant to cefotaxime. Strains with phenotypic resistance to cefotaxime represent strains resistant to beta-lactamase. Phenotypic gentamicin resistance represents phenotypic aminoglycoside resistance, and phenotypic tetracycline resistance represents phenotypic tetracycline resistance. Genotypic trimethoprim/sulphonamide resistance represents the joint component of genotypic sulphonamide resistance and genotypic trimethoprim resistance shown by the strains.

## Data Availability

The whole genome sequence data of 91 *E. coli* strains have been submitted to the NCBI under the BioProject accession number PRJNA1043574.

## Supplementary Materials

The following supporting information can be downloaded at: https://www.mdpi.com/article/10.3390/microorganisms12040653/s1, Table S1: Source information, clermonTyping, plasmid replicons types andST typing of 91 ESBL-EC strains; Table S2: Sensitivity test results of 91 ESBL-EC strains to 10 antibiotics.
